# O-GlcNAcylation of the tumor suppressor LATS1 drives mitotic progression *via* PLK1

**DOI:** 10.1016/j.jbc.2025.110990

**Published:** 2025-12-01

**Authors:** Li Meng, Yunfeng Wang, Wen Zhou, Shian Wu, Jing Li

**Affiliations:** 1 Beijing Key Laboratory of DNA Damage Response and College of Life Sciences, Capital Normal University, Beijing, China; 2 The State Key Laboratory of Medicinal Chemical Biology, Tianjin Key Laboratory of Protein Sciences, College of Life Sciences, Nankai University, Tianjin, China; 3 College of Chemistry and Molecular Engineering, Peking University, Beijing, China

**Keywords:** LATS1, Hippo, OGT, O-GlcNAc, PLK1

## Abstract

Initially discovered in *Drosophila*, the Hippo pathway is pivotal for tissue growth and organ homeostasis. It is regulated by both extrinsic and intrinsic signals and exerts its effect *via* a core kinase cascade, in which large tumor suppressor 1 and 2 (LATS1/2) plays a key role. LATS1 has also been shown to regulate mitotic progression by phosphorylating myosin phosphatase targeting subunit 1 (MYPT1) to counteract the activity of polo-like kinase 1 (PLK1), a mitotic master kinase. Herein, we demonstrate that the hexosamine biosynthetic pathway regulates the Hippo pathway *via* LATS1. We show that LATS1 interacts with the O-GlcNAc transferase (OGT) and is O-GlcNAcylated. *Via* electron transfer dissociation mass spectrometry, we mapped the O-GlcNAcylation sites to be S479/S482/T484/T485. O-GlcNAcylation attenuates LATS1 protein stability and downregulates the phosphorylation level of its downstream substrates, such as MYPT1. Subsequently, decreased MYPT1-pS473 levels enhance PLK1-pT210 levels and drive mitotic progression. Importantly, we demonstrate that in *Drosophila* O-GlcNAcylation of LATS1 promotes the wing size. Thus, this study suggests that O-GlcNAcylation links extrinsic glucose levels to LATS1 in the Hippo pathway and cell proliferation.

The Hippo pathway was initially discovered in *Drosophila* and found to regulate cell growth and tissue homeostasis (1, 2). The core Hippo signaling cascade in mammals include the upstream kinases–mammalian STE20-like protein kinase half (MST1/2) and large tumor suppressor (LATS1/2); nuclear transcription factors–TEA Domain Transcription Factor (TEAD1/2/3/4); and the coactivator Yes-associated protein 1 (YAP) and PDZ-binding motif protein (TAZ) (1, 2). MST1/2 phosphorylates and activates LATS1/2, which in turn phosphorylates and stabilizes YAP for cytoplasmic retention. The MST1/2-LATS1/2-YAP pathway is a tumor suppressor pathway, while the un-phosphorylated YAP is imported into the nucleus where it promotes cell proliferation. The Hippo pathway responds to diverse upstream signals, such as heat shock, osmotic stress, mechanical force and energy stress (1, 2).

O-linked β-N-acetylglucosamine (O-GlcNAc) modification is a nutrient-sensing post-translational modification (PTM) that integrates various metabolites into signaling pathways (3). O-GlcNAc intersects with the Hippo pathway. O-GlcNAc transferase (OGT) modifies YAP at S109, which disrupts the interaction between YAP and LATS1, resulting in unphosphorylated YAP and transcriptional activation (4). In high-glucose-stimulated liver tumorigenesis, YAP O-GlcNAcylation was identified to be at T241, which also led to YAP unphosphorylation and attenuated the pro-tumorigenic capacities of YAP (5). In diabetic retinopathy, YAP was O-GlcNAcylated at T383, which antagonizes YAP phosphorylation, thus promoting vascular dysfunction (6). Besides YAP, the Ser/Thr kinase LATS2 is also O-GlcNAcylated. O-GlcNAc at T436 disrupts the interaction between LATS2 and its adaptor protein, suppresses LATS2 kinase activity and activates YAP (7). In OGT-knockout livers, aneuploidy phenotypes were discernable with disruptions in many pathways, including Hippo (8). Conversely, OGT itself is a YAP target gene (4).

In this study, we identify that LATS1 is O-GlcNAcylated. LATS1 is a tumor suppressor in the Hippo core kinase cascade (9). It transduces signals from upstream MST1/2 to downstream YAP/TAZ and plays a role in cancer immunity (10, 11), autophagy (12) and mitosis (13, 14, 15). During mitotic progression, LATS1 was shown to phosphorylate myosin phosphatase-targeting subunit 1 (MYPT1) at S445 (also at S473/S507/T508/S509) (13). As MYPT1 is a negative regulator of the mitotic master kinase polo-like kinase 1 (PLK1) (16), LATS1 thus activates PLK1 and promotes mitotic progression (13).

Here, using electron transfer dissociation (ETD) mass spectrometry (MS), we identify that LATS1 is O-GlcNAcylated at S479/S482/T484/T485. O-GlcNAcylation attenuates LATS1 stability and decreases MYPT1 phosphorylation at S473, thus elevating PLK1 activity and promoting mitotic progression. More importantly, using transgenic flies, we show that LATS1 O-GlcNAcylation upregulates the wing size in *Drosophila*. Taken together, our findings reveal that LATS1 O-GlcNAcylation plays a pivotal role in mitosis and cell proliferation by regulating PLK1.

## Results

### LATS1 interacts with OGT and undergoes O-GlcNAcylation

**Figure 1:**
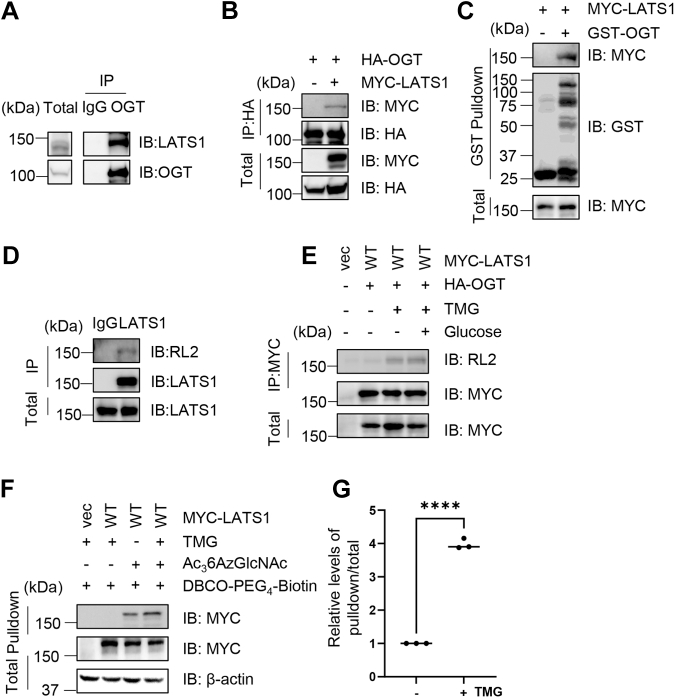
**LATS1 is O-GlcNAcylated.***A*, endogenous LATS1 interacts with OGT. Cellular lysates were immunoprecipitated with anti-OGT antibodies and immunoblotted with the antibodies indicated. *B*, 293T cells were transfected with MYC-LATS1 and HA-OGT plasmids. Cell lysates were subjected to immunoprecipitation followed by immunoblotting with the indicated antibodies. *C*, 293T cells were transfected with MYC-LATS1 plasmids, and cell lysates were incubated with recombinant GST-OGT proteins for GST pull-down assays. *D*, cellular lysates were immunoprecipitated with anti-LATS1 antibodies, and the immunoprecipitates were subject to immunoblotting using the antibodies as indicated. *E*, 293T cells were treated with the OGA inhibitor Thiamet-G (TMG) plus glucose as previously described (1). Cell lysates were immunoblotted with the anti-O-GlcNAc antibody RL2. *F*, 293T cells transfected with MYC-LATS1 plasmids were treated as previously described (2): with or without 200 μmol/L Ac_3_6AzGlcNAc, and with or without 5 μmol/L TMG. *G*, quantitative analysis of (F). ∗∗ indicates a statistically significant difference by one-way ANOVA (n = 3; ∗∗, *p* < 0.01).

To examine whether LATS1 is O-GlcNAcylated, we first investigated its interaction with OGT. Co-immunoprecipitation (co-IP) using an anti-OGT antibody followed by immunoblotting (IB) revealed that endogenous LATS1 co-precipitated with OGT (Fig. 1*A*). To validate this interaction with exogenous overproduced proteins, we co-transfected 293T cells with HA-OGT and MYC-LATS1 plasmids. Anti-HA co-IP showed their association (Fig. 1*B*). Further pull-down assays using recombinant GST-OGT proteins pulled down MYC-LATS1 proteins from cellular lysates (Fig. 1*C*), suggesting that LATS1 interacts with OGT.

We next examined whether LATS1 is O-GlcNAcylated. First, cellular lysates were subject to IP with anti-LATS1 antibodies, then the immunoprecipitates were IBed with anti-RL2 antibodies (a pan-O-GlcNAc antibody). And the results showed that endogenous LATS1 was O-GlcNAcylated (Fig. 1*D*). We then enriched for O-GlcNAcylation by treating the cells with the O-GlcNAcase inhibitor Thiamet-G (TMG) and glucose (Glu) as previously described (17), and the result showed robust O-GlcNAc signals on immunoprecipitated LATS1 (Fig. 1*E*). We also found LATS1 O-GlcNAcylation in click chemistry assays (Fig. 1, *F* and *G*). These results demonstrate that LATS1 interacts with OGT and is O-GlcNAcylated.

### LATS1 is O-GlcNAcylated at Ser479/Ser482/Thr484/Thr485

**Figure 2:**
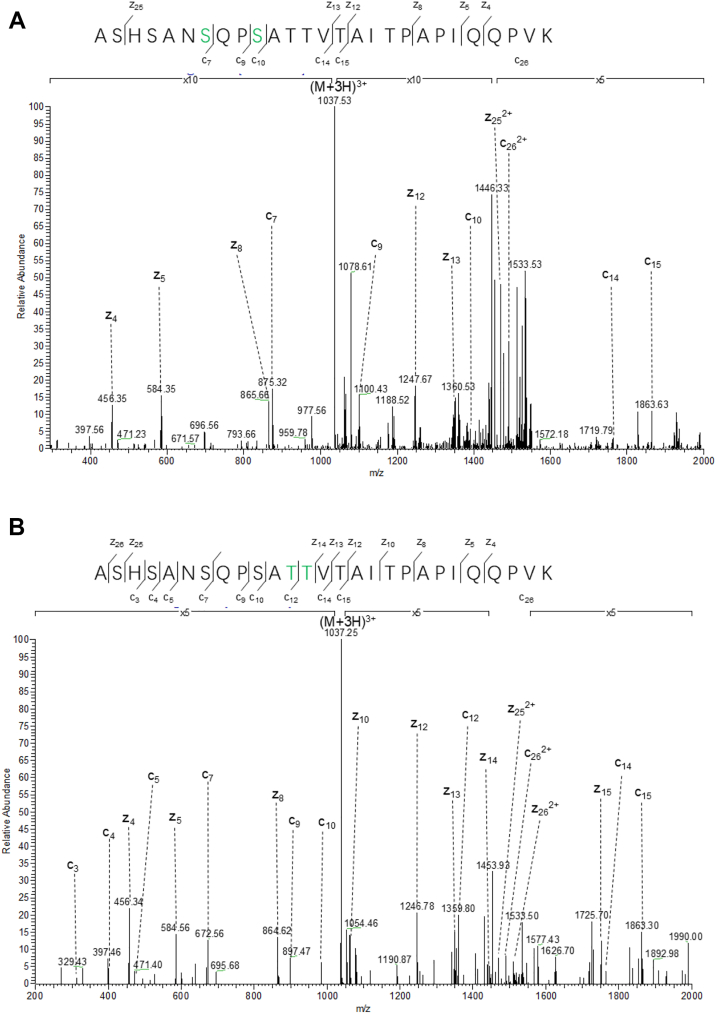
**ETD mass spectrometry identification of O-GlcNAcylation sites at S479/S482/T484/T485 on LATS1.***A–B*, 293T cells transfected with MYC-LATS1 were subjected to O-GlcNAc enrichment. Anti-MYC immunoprecipitates were analyzed by ETD mass spectrometry, revealing four putative O-GlcNAcylation sites: Ser479/Ser482/Thr484/Thr485.

To map the O-GlcNAcylation sites, we transfected 293T cells with MYC-LATS1, immunoprecipitated the protein and analyzed it by ETD MS. Modified peptides were identified, with fragmentation patterns pinpointing Ser479, Ser482, Thr484, and Thr485 as O-GlcNAc sites (Fig. 2, *A* and *B*) (Supplementary Table S1).

**Figure 3:**
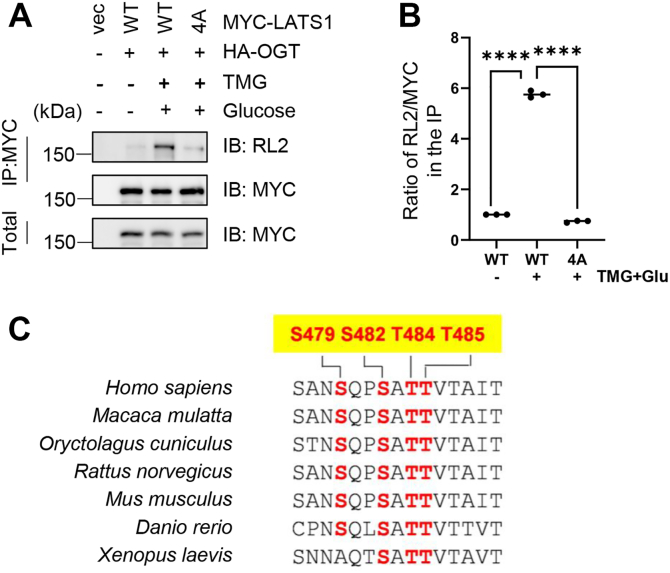
**Mutational analysis identifies critical O-GlcNAcylation sites on LATS1.***A*, immunoblotting analysis of O-GlcNAc-modified LATS1 in 293T cells expressing either wild-type (WT) or the quadruple alanine mutant (4A: S479A/S482A/T484A/T485A). Lysates were immunoprecipitated with anti-LATS1 antibodies and probed with anti-O-GlcNAc (RL2) and anti-LATS1 antibodies. *B*, quantification of O-GlcNAcylation levels from three independent experiments (mean ± SEM). ∗∗∗∗*p* < 0.0001 by one-way ANOVA. *C*, multiple sequence alignment demonstrating evolutionary conservation of the identified O-GlcNAcylation sites (highlighted in *red*) across indicated species.

To validate these sites, we generated a quadruple alanine mutant (S479A/S482A/T484A/T485A, 4A) of LATS1. Cells expressing MYC-LATS1-WT or MYC-LATS1-4A were treated with TMG + Glu, and the 4A mutation markedly reduced O-GlcNAc signals as detected by the RL2 antibody (Fig. 3, *A* and *B*). Sequence alignment showed that these residues of LATS1 are highly conserved in vertebrates (Fig. 3*C*), but not in invertebrates. Thus, major LATS1 O-GlcNAcylation sites include Ser479, Ser482, Thr484, and Thr485, though other sites may exist.

### O-GlcNAcylation of LATS1 promotes its ubiquitination

**Figure 4:**
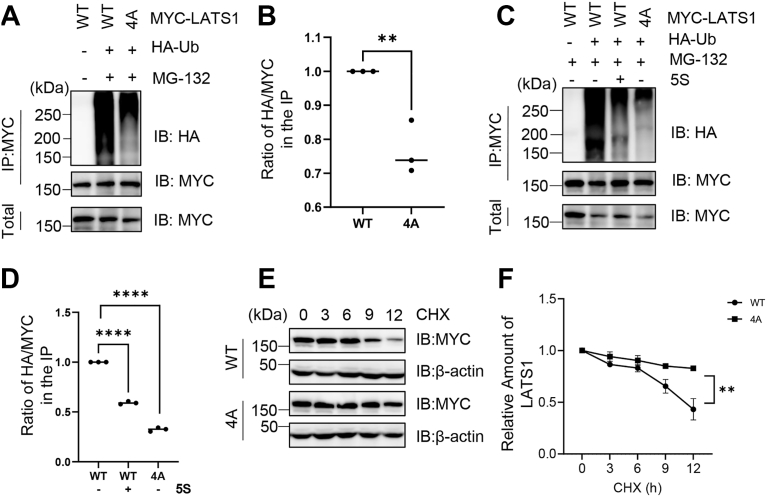
**O-GlcNAcylation of LATS1 promotes its ubiquitination.***A*, 293T cells were transfected with LATS1-WT or LATS1-4A mutants, along with HA-Ub plasmids and treated with 10 μM MG-132 (a proteasome inhibitor). Cell lysates were immunoprecipitated with an anti-MYC antibody and analyzed by immunoblotting using the indicated antibodies. *B*, quantification of (*A*). ∗∗ indicates a statistically significant difference by one-way ANOVA (n = 3; ∗∗, *p* < 0.01). *C*, 293T cells were transfected with LATS1-WT or LATS1-4A mutants, along with HA-Ub plasmids, treated with 10 μM MG-132, and then either exposed to an OGT inhibitor Acetyl-5S-GlcNAc (5S) or left untreated. *D*, quantification of (*C*). ∗∗∗∗indicates a statistically significant difference by one-way ANOVA (n = 3; ∗∗∗∗, *p* < 0.0001). (*E*, *F*) Cycloheximide (CHX) pulse-chase assay. Cells transfected with LATS1-WT or LATS1-4A plasmids were treated with CHX for the indicated durations. *F*, shows the quantification. Statistical significance was determined by two-way ANOVA (n = 3; ∗∗, *p* < 0.01).

As O-GlcNAcylation often regulates protein stability (18), we hypothesized that this modification may modulate LATS1 ubiquitination. Cells were co-transfected with LATS1-WT or LATS1-4A with HA-Ub plasmids, followed by proteasome inhibition (MG-132 treatment). The results revealed significantly reduced ubiquitination of the 4A mutant (Fig. 4, *A* and *B*). Consistently, treatment with the OGT inhibitor Acetyl-5S-GlcNAc (5S) diminished LATS1 ubiquitination (Fig. 4, *C* and *D*). Cycloheximide (CHX) pulse-chase assays further demonstrated faster degradation of LATS1-WT compared to the 4A mutant (Fig. 4, *E* and *F*). Together, these data indicate that O-GlcNAcylation promotes LATS1 ubiquitination.

### O-GlcNAcylation of LATS1 regulates phosphorylation of MYPT1 and PLK1

**Figure 5:**
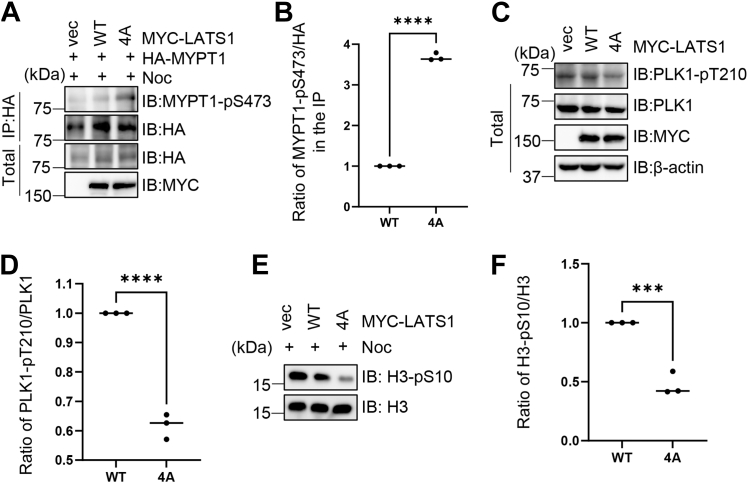
**O-GlcNAcylation of LATS1 regulates phosphorylation of MYPT1 and PLK1.***A*, 293T cells were co-transfected with LATS1-WT or LATS1-4A plasmids together with HA-MYPT1, followed by treatment with nocodazole (noc). Cell lysates were subject to immunoprecipitation using an anti-HA antibody, followed by immunoblotting with the indicated antibodies. *B*, quantification of (*A*). ∗∗∗∗ indicates a statistically significant difference by one-way ANOVA (n = 3; ∗∗∗∗, *p* < 0.0001). *C*, 293T cells transfected with LATS1-WT or LATS1-4A were lysed and analyzed by immunoblotting the antibodies indicated. *D*, Quantification of (*C*). ∗∗∗∗ indicates a statistically significant difference by one-way ANOVA (n = 3; ∗∗∗∗, *p* < 0.0001). *E*, histones were extracted from 293T cells expressing LATS1-WT or LATS1-4A and immunoblotted with anti–H3-pS10 antibody. *F*, quantification of H3-pS10 levels in (*E*). ∗∗∗ indicates a statistically significant difference by one-way ANOVA (n = 3; ∗∗∗, *p* < 0.001).

The Hippo pathway is known to be interlinked with mitosis (1, 19). Specifically, LATS1 phosphorylates MYPT1 to modulate PLK1-Thr210 phosphorylation (13). To test whether LATS1 O-GlcNAcylation has any effects on mitosis, we co-transfected LATS1-WT or LATS1-4A with HA-MYPT1 plasmids into cells. The cells were subsequently arrested in mitosis *via* nocodazole (noc) treatment. And we observed that the 4A mutant showed more robust MYPT1-Ser473 phosphorylation signals (Fig. 5, *A* and *B*), which might be due to elevated protein level of LATS1-4A.

As MYPT1-pS473 is known to downregulate PLK1-pT210 (16), a pivotal activation phosphorylation of PLK1, we next sought to determine the effect of 4A on PLK1-pT210. As shown in Figure 5, *C* and *D*, PLK1-pT210 levels were attenuated upon LATS1-4A transfection, which is consistent with Figure 5, *A* and *B*.

Furthermore, histone extraction from LATS1-WT or LATS1-4A-transfected cells revealed decreased H3 Ser10 phosphorylation (a mitotic marker) in the 4A mutant (Fig. 5, *E* and *F*), suggesting S10-histone phosphorylation is lower. Thus, LATS1 O-GlcNAcylation regulates MYPT1 and PLK1 phosphorylation to promote cellular proliferation.

### O-GlcNAcylation fine-tunes LATS1 activity in *Drosophila*

Warts/Lats (Wts), the *Drosophila* homologue of mammalian LATS1, is a core component of the Hippo signaling pathway. Loss-of-function of Wts resulted in a range of developmental defects, including tissue overgrowth (20, 21), and all these defects could be rescued by human LATS1 (22), indicating the fundamental function of Wts is highly conserved.

**Figure 6:**
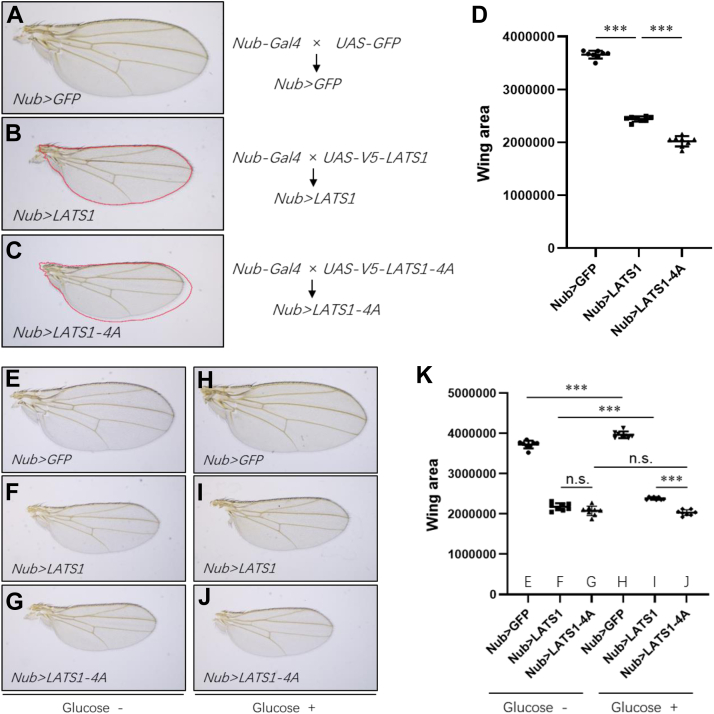
**O-GlcNAcylation of LATS1 regulates the wing size in *Drosophila*.***A–C*, flies were crossed and maintained in vials containing standard medium. (*A*) *Nub-Gal4* crossed with *UAS-GFP* as a control; (*B*) *LATS1*, and (*C*) *LATS1-4A* overexpression driven by *Nub-Gal4* in *Drosophila* wings, respectively. *D*, Wing areas of the genotypes in (*A*–*C*) were measured. Differences among normally distributed values were analyzed by the unpaired *t* test (n = 8; ∗∗∗, *p* < 0.001). *E–J*, flies were crossed and maintained in vials containing no glucose medium (*E*–*G*) or standard medium (*H*–*J*). (*E*) and (*H*) *Nub-Gal4* crossed to *UAS-GFP*; (*F*) and (*I*) *LATS1* overexpression driven by *Nub-Gal4*; (*G*) and (*J*) *LATS1-4A* overexpression driven by *Nub-Gal4*. *K*, Wing areas of the genotypes in *E*–*J* were measured. Differences among normally distributed values were analyzed by the unpaired *t* test (n = 8; n.s., not significant; ∗∗∗, *p* < 0.001).

Since the O-GlcNAcylation sites of LATS1 are not conserved in Wts, we generated transgenic lines to explore the functional relevance of human LATS1 O-GlcNAcylation in *Drosophila*. In these lines, LATS1-4A is expressed at the same level as wild type (Fig. S1). Similar to the small wing phenotype caused by Wts overexpression using the *Nub-Gal4* driver (*Nub > Wts*) (23), *Nub > LATS1* also led to a small wing phenotype (Fig. 6, *A*, *B* and *D*). Intriguingly, the wing size was reduced more by overexpression of the O-GlcNAcylation-defective mutant LATS1-4A (*Nub > LATS1-4A*) (Fig. 6, *A*–*D*), suggesting LATS1 activity is suppressed by O-GlcNAcylation.

To further evaluate whether LATS1 responds to glucose availability, *Nub > LATS1* and *Nub > LATS1-4A* flies were reared on medium supplied with or without glucose. Our results showed that glucose enhanced the wing size of the control (GFP overexpression/*Nub > GFP*) (Fig. 6, *E*, *H* and *K*) and LATS1 overexpression (*Nub > LATS1*) (Fig. 6, *F*, *I* and *K*) flies. However, the wing size of *Nub > LATS1-4A* is no longer responding to glucose (Fig. 6, *G*, *J* and *K*). In addition, flies of *Nub > LATS1* and *Nub > LATS1-4A* showed the same wing size (Fig. 6, *F*, *G* and *K*) under glucose deprivation, but *Nub > LATS1* flies had larger wings than *Nub > LATS1-4A* when glucose was supplied (Fig. 6, *I*–*K*), further supporting the notion that O-GlcNAcylation fine-tunes LATS1 activity *in vivo*. Taken together, glucose-mediated O-GlcNAcylation represses LATS1 activity and thus enhances the wing size.

## Discussion

**Figure 7:**
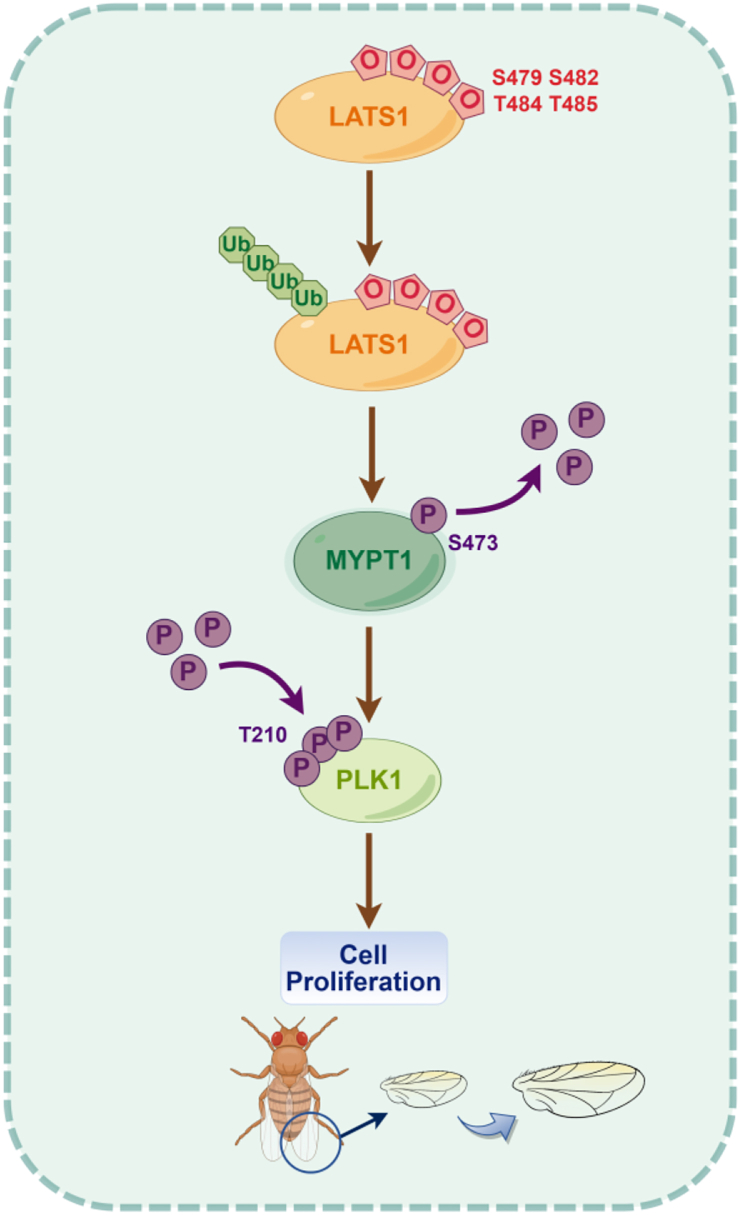
**A model depicting the role of LATS1O-GlcNAcylationin mitosis.** We propose that O-GlcNAcylation of LATS1 at residues S479/S482/T484/T485 promotes its ubiquitination. Degraded LATS1 thus downregulates MYPT1 phosphorylation at Ser473, enhances PLK1 phosphorylation at Thr210, and ultimately drives cell proliferation. By FigDraw.

In this work, we present evidence that LATS1 is O-GlcNAcylated at S479/S482/T484/T485, which promotes its ubiquitination and degradation, attenuates MYPT1-Ser473 phosphorylation, enhances PLK1 Thr210 phosphorylation and activation, and ultimately accelerates cell proliferation (Fig. 7).

In our MS analysis, we used ETD fragmentation directly on abundant MS1 peptides, as we believe this approach is more efficient. While Higher-energy collisional dissociation (HCD) can confirm the presence of O-GlcNAc modification through the characteristic neutral loss, it does not retain the modification on fragment ions, thereby limiting its ability to provide precise site assignment—especially for peptides carrying multiple O-GlcNAc modifications. In contrast, ETD preserves the labile modification and allows simultaneous peptide sequencing and site localization. That being said, greater confidence would occur if HCD were done first to select peptides showing the diagnostic loss of GlcNAc. It is also possible that LATS1 may contain other O-GlcNAcylation sites.

It is interesting that LATS1 O-GlcNAc sites are not conserved in *Drosophila* (Fig. 3*C*). And it is the same case for ADP-ribose glycohydrolase (PARG) (24). O-GlcNAcylation regulates the subcellular localization of human PARG. *Drosophila* PARG O-GlcNAc sites *per se* are not conserved, but they are close to a putative nuclear localization signal, just like their human homolog. Thus, it is possible that *Drosophila* LATS1 is also O-GlcNAcylated, which has a conserved role in enhancing ubiquitination.

We did examine the effect of LATS1 O-GlcNAcylation on LATS1-pT1079 and YAP-pS127 levels, but no discernable effects were observed. The Hippo pathway is homologous to the yeast mitotic exit network (MEN) pathway, and both LATS1 and YAP regulate mitotic progression (14). Specifically, LATS1 modulates Cdc25B stability and suppresses centrosome overduplication (25), and regulates G2/M checkpoint (26, 27). *Drosophila* WARTS is phosphorylated by CDK1 and localizes to the mitotic spindle (28). YAP has a non-transcriptional function in mitosis, as it is phosphorylated by CDK1 (29), regulates the spindle checkpoint (30), colocalizes with the mitotic spindle and midbody in lung cancer cells (31), is essential for the proper organization of the cytokinesis apparatus (32), and induces mitotic rounding in adult cardiomyocytes (33). Thus, the O-GlcNAc sites we identified on LATS1 may not directly pertain to the canonical transcriptional role of YAP, which is evidenced by YAP-pS127 levels. Because O-GlcNAc is known to occur on multiple residues, it is conceivable that other O-GlcNAc sites on LATS1 may directly regulate YAP in transcription.

Besides O-GlcNAcylation, LATS1 is also subject to other PTMs. Upon interferon (IFN) treatment, LATS1 is phosphorylated at Tyr200 by Tyrosine kinase 2 (Tyk2) for activation and nuclear import, subsequently leading to YAP degradation and IFN-I antiviral activity (34). LATS1 is acetylated at K751 by CBP for stabilization and inactivation, resulting in YAP nuclear translocation and gene transcription (35). Intriguingly, LATS1 is phosphorylated at S464 by AMPK-related protein kinase 5 (ARK5 or NUAK1), which regulates LATS1 stability (36). NUAK1 promotes senescence by diminishing LATS1 and is implicated in cellular senescence and cellular ploidy (36). As S464 is proximal to the O-GlcNAcylation sites, it is a possible scenario that LATS1 O-GlcNAcylation has crosstalk with pS464 to mediate LATS1 abundance. It is also worth mentioning that the site mapping were performed using asynchronous cells. Our results showed that there are still some O-GlcNAc signals in the 4A mutant cells (Fig. 3*A*). As O-GlcNAc is known to respond to stress and stimuli, we suspect that there could be more LATS1 O-GlcNAc sites at M phase.

In conclusion, we identified a new OGT substrate in the Hippo pathway that drives cell proliferation. Due to the conserved role of LATS1 in suppressing tumorigenesis, we speculate that distinct LATS1 O-GlcNAc sites will be identified in different types of cancer in the future. We also envision that O-GlcNAc interplays with other components of the Hippo pathway to exert disparate physiological functions.

## Experimental procedures

### Cell culture, antibodies, and plasmids

293T cells were obtained from the American Type Culture Collection (ATCC). All cell lines utilized in this study were validated *via* short tandem repeat (STR) profiling and confirmed free of *mycoplasma* contamination before experiments. The following antibodies were used: anti-Myc (PTM Bio, # PTM-5390), anti-RL2 (Abcam, #ab2739), anti-GST (Gene Script, # A00865), anti-HA (DIA-An, # 3063), anti-OGT (Abcam, # ab96718), anti-LATS1 (Cell Signaling Technology, # 3477S), anti-PLK1 (Santa Cruz Biotechnology, # SC-17783), anti-H3-pS10 (Bethyl, #A301-844A), anti-H3 (Abcam, #ab4729), anti-β-actin (Sigma, Cat.# A5441), and anti-PLK1-pT210 (Abcam, Cat.# ab39068). Antibodies against pSer-473 were described before (17, 37).

LATS1 mutant plasmids were generated using the QuickChange II Site-Directed Mutagenesis Kit (Stratagene) according to the manufacturer’s protocols, with specific primer sequences available upon request. The fly expression plasmids for V5-tagged LATS1 and V5-LATS1-4A were generated by PCR from pcDNA3.0-Myc-LATS1 and pcDNA3.0-Myc-LATS1-4A, and cloned into the pUASTattB vector, respectively. All plasmids generated in this study were confirmed by DNA sequencing.

### Chemical treatments

Cells were treated with nocodazole (Noc) at a final concentration of 330 nM for 16 h. 5S (OGT inhibitor) was used at a final concentration of 50 μM for 24 h. Thiamet-G (TMG) was used at 0.25 mM for 24 h. Glucose was used at 30 mM for 3 h. MG-132 was used at 10 μM for 12 h.

### Immunoprecipitation and immunoblotting assays

Immunoprecipitation and immunoblotting were performed as previously described (38, 39). The primary antibodies used for immunoblotting were as follows: anti-HA (1:5000), anti-RL2 (1:1000), anti-OGT (1:1000), anti-Myc (1:2000), anti-GST (1:3000), anti-β-actin (1:3000), anti-LATS1 (1:1000), anti-PLK1 (1:500), anti-PLK1-pT210 (1:1000), and anti-MYPT1-pS473 (1:500). Horseradish peroxidase-conjugated secondary antibodies were purchased from Jackson ImmunoResearch. Immunoreactive bands were detected using an enhanced chemiluminescence (ECL) system (Amersham) and visualized with a LAS-4000 imaging system (Fujifilm). Densitometric quantification was performed using Multi Gauge software (Fujifilm), and all Western blot experiments were repeated a minimum of three times.

### Biorthogonal chemistry assays

Biorthogonal chemistry experiments were performed as described (40). Cells were treated with 200 μmol/L Ac_36_AzGlcNAc for 24 h, then harvested and lysed in 150 mM lysis buffer (150 mM NaCl, 1 M Tris-HCl, pH 7.5, 0.5 M EDTA, 10% NP-40) containing a protease inhibitor cocktail (Roche) at 4 °C for 1 h. Following centrifugation (4 °C, 12,000×*g*, 10 min) to clarify the lysate, the supernatant was reacted with 50 μmol/L DBCO-PEG4-biotin (Duyouyou Biotechnology), 8 mmol/L urea, 10 mmol/L Hepes (pH 7.9), and Halt Protease and Phosphatase Inhibitor Cocktail (100 × , Thermo Fisher Scientific). The final pull-down complexes isolated by streptavidin magnetic beads were subjected to Western blotting analysis.

### ETD mass spectrometry

#### Sample preparation:

The gel band pieces were dehydrated in acetonitrile, incubated in 10 mM DTT in 50 mM ammonium bicarbonate at 56 °C for 40 min, incubated in 55 mM iodoacetamide in 50 mM ammonium bicarbonate at ambient temperature for 1 h in the dark, and dehydrated again. Then the gel pieces were digested in-gel with 2 ng/μl sequencing grade trypsin in 50 mM ammonium bicarbonate overnight at 37 °C. The resulting peptides were extracted twice with 5% formic acid/50% acetonitrile, then vacuum-centrifuged to dryness. All samples were resuspended in 0.1% FA in water prior to LC-MS/MS analysis.

#### LC-MS/MS parameters

Peptides were separated using a loading column (100 μm × 2 cm) and a C18 separating capillary column (75 μm × 15 cm) packed in-house with Luna 3 μm C18(2) bulk packing material (Phenomenex). The mobile phases (A: water with 0.1% formic acid and B: 100% acetonitrile with 0.1% formic acid) were driven and controlled by an EASY-nLC 1000 system (Thermo Fisher Scientific). The LC gradient was held at 2% for 1 min of the analysis, followed by an increase from 2% to 7% B from 1 to 2 min, an increase from 7% to 35% B from 2 to 62 min, and an increase from 35% to 75% B from 62 to 66 min.

The MS data were acquired in data-dependent mode with a full MS scan (300–1700 M/z) in FT mode at a resolution of 60,000 followed by ETD MS/MS scans on the 10 most abundant ions with multiple charges in the initial MS scan. Automatic gain control (AGC) targets were 1e6 ions for Orbitrap scans and 5e4 for MS/MS scans. For dynamic exclusion, the following parameters were used: isolation window, 2 m/z; repeat count, 1; repeat duration, 25 s; and exclusion duration, 25 s. The ETD activation time was 150 ms. Charge state dependent time and supplemental activation for ETD were enabled.

#### Data analysis

Data processing was carried out using Thermo Proteome Discoverer 2.4 using a SwissProt homo sapiens database (TaxID = 9606 and subtaxonomy, 42,253 protein sequences). Carbamidomethyl (Cys) were chosen as a static modification, oxidation (Met) and HexNAc (Ser or Thr) were chosen as variable modifications. Mass tolerance was 10 ppm for precursor ions and 0.6 Da for fragment ions. Maximum missed cleavages was set as 2. Peptide spectral matches (PSM) were validated using the Percolator algorithm, based on q-values at a 1% FDR at both peptide and protein level.

### Histone extraction

The cell pellet was resuspended in 500 μl of TEB buffer (1×PBS containing 0.5% Triton X-100) supplemented with protease inhibitor cocktail (PIC) at a 9:1 ratio, followed by incubation on ice for 10 min for lysis. After lysis, the sample was centrifuged at 2000 rpm for 10 min at 4 °C, and the supernatant was discarded. The pellet was resuspended in 500 μl of the same TEB/PIC mixture and centrifuged again under identical conditions (2000 rpm, 10 min, 4 °C). The resulting pellet was incubated with 100 μl of 0.2 M HCl at 4 °C for 12 h to extract histones. Following centrifugation, the supernatant was neutralized by adding one-fifth volume of 1 M NaOH, mixed with an appropriate amount of sample buffer, and boiled for further analysis.

### Fly genetics

The pUASTattB plasmids were microinjected individually into fly embryos to get the transgenic flies for V5-LATS1 or V5-LATS1-4A overexpression induced by Nub-Gal4. The standard cornmeal-based medium was prepared in 3 L batches containing: 3 L deionized water, 240 g instant dry yeast (Angel), 330 g glucose, 120 g powdered cornmeal, and 16.5 g bacteriological agar (Solarbio, # A8190). The agar-nutrient mixture was boiled for 25 min with continuous stirring, then cooled to 65 °C. At this stage, an antifungal solution—containing 5.1 g ethyl 4-hydroxybenzoate (Aladdin, #E105142-500g) pre-dissolved in 50 ml of 95% (v/v) ethanol (10.2% w/v concentration)—was thoroughly incorporated into the medium prior to solidification. The glucose-free medium was prepared as the standard medium except no glucose was supplied.

## Data availability

The mass spectrometry proteomics data have been deposited to the ProteomeXchange Consortium *via* the PRIDE (41) partner repository with the dataset identifier PXD064782 and 10.6019/PXD064782.

## Supporting information

This article contains supporting information.

## Conflict of interest

The authors declare that they have no conflicts of interest with the contents of this article.
