# Supplementary material for: O-GlcNAcylation of the tumor suppressor LATS1 drives mitotic progression via PLK1
Source: J Biol Chem. 2025 Dec 1;302(1):110990. doi: 10.1016/j.jbc.2025.110990 (PMC12797046; doi:10.1016/j.jbc.2025.110990)
Supplement: Supporting Figure S1 [file mmc2.pptx]

## Slide 1
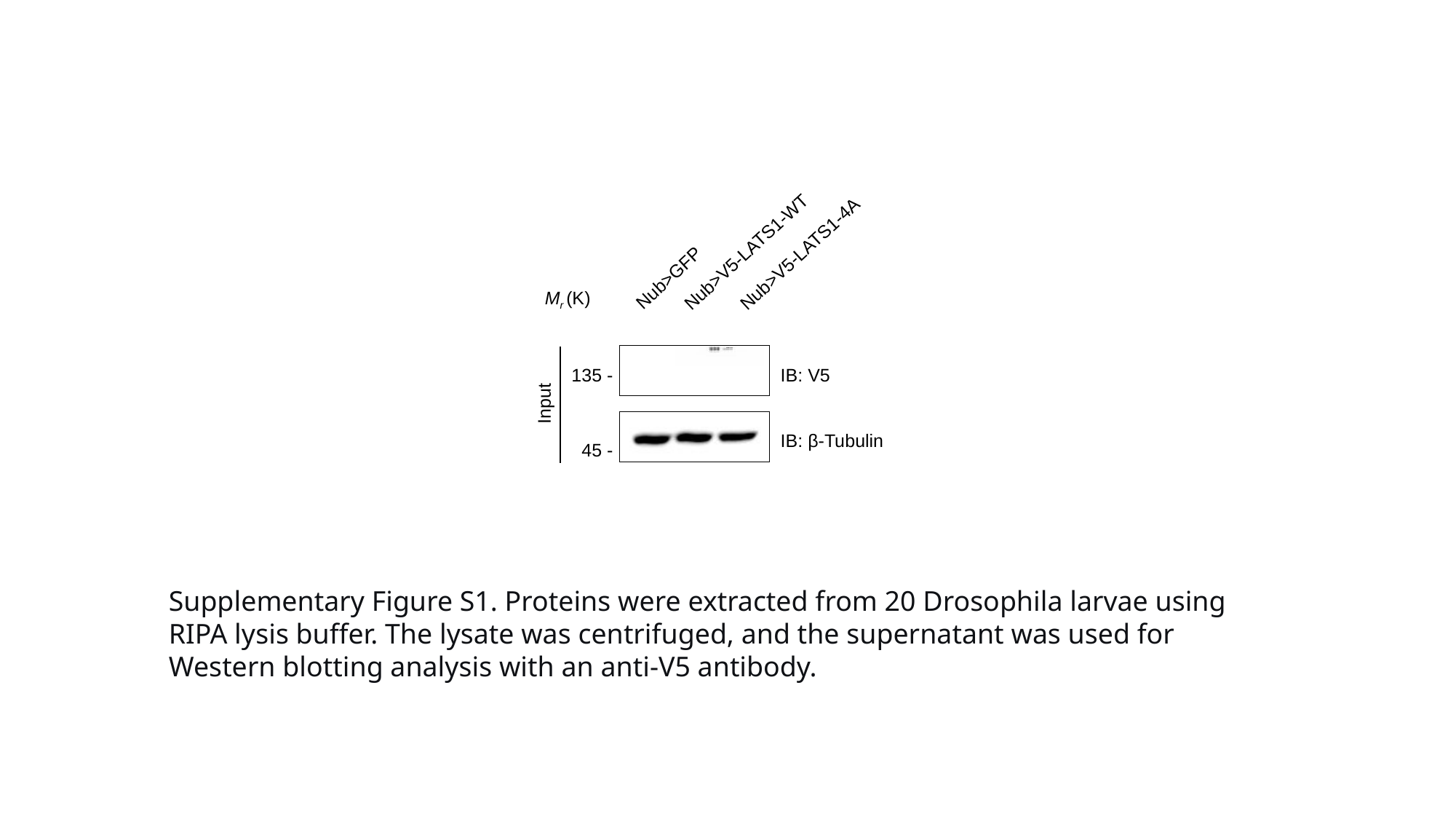

Nub>GFP
Nub>V5-LATS1-WT
Nub>V5-LATS1-4A
Mr (K)
135 -
IB: V5
Input
IB: β-Tubulin
45 -
Supplementary Figure S1. Proteins were extracted from 20 Drosophila larvae using RIPA lysis buffer. The lysate was centrifuged, and the supernatant was used for Western blotting analysis with an anti-V5 antibody.
